# Supplementary material for: Production of Promising Heat-Labile Enterotoxin (LT) B Subunit-Based Self-Assembled Bioconjugate Nanovaccines against Infectious Diseases
Source: Vaccines (Basel). 2024 Mar 23;12(4):347. doi: 10.3390/vaccines12040347 (PMC11054625; doi:10.3390/vaccines12040347)
Supplement: Supplementary file 1 [file vaccines-12-00347-s001.zip › vaccines-2911689-supplementary.pdf]

Supplementary Material

Supplementary Table S1：Endotoxin level of the *Salmonella paratyphi* A nanovaccines.

Table S1：Endotoxin level of the *Salmonella paratyphi* A nanovaccines.

Sample ID	Endotoxin (EU/mL)
PBS	7.5±0.007
NP(LTIB)-OPS_{SPA}	16.7±0.011
NP(LTIIbB)-OPS_{SPA}	23.6±0.004

Supplementary Table S2：Endotoxin level of the *Shigella flexneri* 2a nanovaccines.

Table S3：Endotoxin level of the *Shigella flexneri* 2a nanovaccines.

Sample ID	Endotoxin (EU/mL)
NP(LTIB)-OPS_{Kp}	18.5±0.003
NP(LTIIbB)-OPS_{Kp}	22.7±0.005

Supplementary Table S3：Endotoxin level of the *Klebsiella pneumoniae* O2 serotype nanovaccines.

Table S3：Endotoxin level of the *Klebsiella pneumoniae* O2 serotype nanovaccines.

Sample ID	Endotoxin (EU/mL)
NP(LTIB)-OPS_{Sf}	22.8±0.006
NP(LTIIbB)-OPS_{Sf}	22.5±0.016

Supplementary Figure S1: The whole blot (uncropped blots) of Figure 1B.

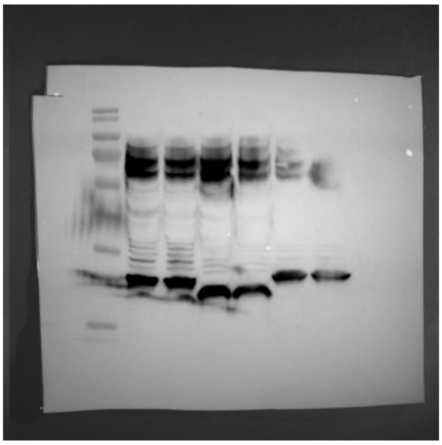

Analysis of the glycoproteins by HRP-conjugated anti-6×His tag antibodies. From left to right, lane 1 is a protein ladder - from top to bottom - 140, 115, 80, 65, 50, 40, 30, 25, 15, 10 kDa. Lane 5 is 50973DWL/LT2 containing the plasmid pPglL-LTIIbBTri with IPTG induction, and the integrated density of bands was 37864.236. Lane 6 is 50973DWL/LT2 containing the plasmid pPglL-LTIBTri with IPTG induction, and the integrated density of bands was 19630.555. Other lanes were not relevant to this study.

Supplementary Figure S2: The whole blot (uncropped blots) of Figure 1E.

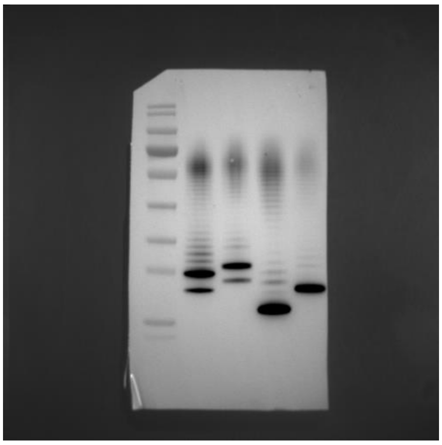

Analysis of the glycoproteins by HRP-conjugated anti-6×His tag antibodies. From left to right, lane 1 is a protein ladder - from top to bottom - 140, 115, 80, 65, 50, 40, 30, 25, 15, 10 kDa, lane 3 is glycoprotein LTIBTri-OPS$_{SPA}$ and lane 5 is glycoprotein LTIIbBTri-OPS$_{SPA}$ were separated by SDS-PAGE and transferred to nitrocellulose membranes for Western Blot analysis using HRP-conjugated anti-6 × His tag antibodies. The integrated density of bands was 24534.884 and 16297.789 respectively. Other lanes were not relevant to this study.

Supplementary Figure S3: The whole blot (uncropped blots) of Figure 1E.

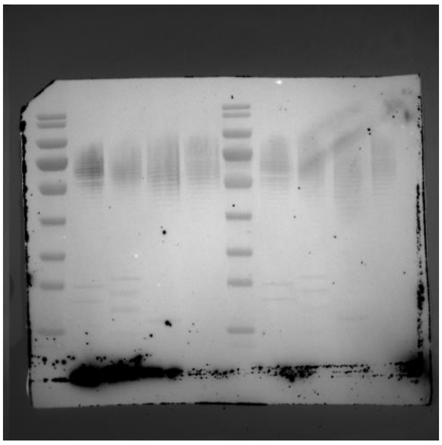

From left to right, lane 6 is protein ladder - from top to bottom - 140, 115, 80, 65, 50, 40, 30, 25, 15, 10 kDa. Lane 8 is glycoprotein LTIBTri-OPS_{SPA} and lane 10 is glycoprotein LTIIbBTri-OPS_{SPA} were separated by SDS-PAGE and transferred to nitrocellulose membranes for Western Blot analysis using rabbit anti *S. paratyphi* A CMCC strain 50973 OPS serums. The integrated density of bands was 34141.458 and 182241.919 respectively. Other lanes were not relevant to this study.

Supplementary Figure S4: The whole blot (uncropped blots) of Figure 5A.

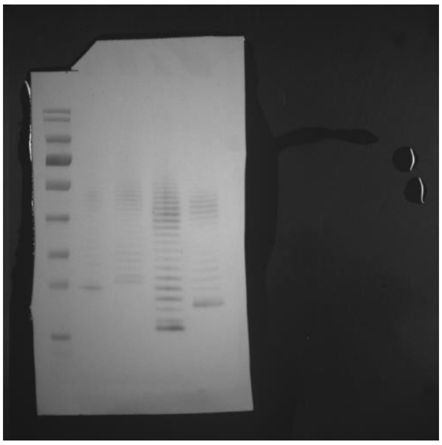

From left to right, lane 1 is protein ladder - from top to bottom - 140, 115, 80, 65, 50, 40, 30, 25, 15, 10 kDa. Lane 3 is nanovaccines NP(LTIB)-OPS_{Sf} and lane 5 is nanovaccines NP(LTIIbB)-OPS_{Sf} pellets were separated by SDS-PAGE and transferred to nitrocellulose membranes for Western Blot analysis using HRP-conjugated anti-6×His tag antibodies. The integrated density of bands was 11452.294 and 19275.092 respectively. Other lanes were not relevant to this study.

Supplementary Figure S5: The whole blot (uncropped blots) of Figure 5A.

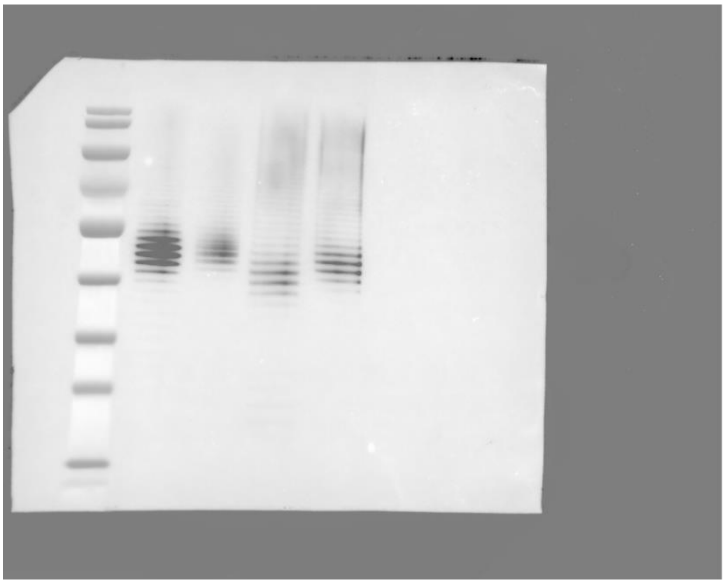

From left to right, lane 1 is protein ladder - from top to bottom - 140, 115, 80, 65, 50, 40, 30, 25, 15, 10 kDa. Lane 3 is nanovaccines NP(LTIB)-OPS_{Sf} and lane 5 is nanovaccines NP(LTIIbB)-OPS_{Sf} pellets were separated by SDS-PAGE and transferred to nitrocellulose membranes for Western Blot analysis using rabbit anti *S. flexneri* 2a strain 301 OPS serums. The integrated density of bands was 27389.052 and 33547.449 respectively. Other lanes were not relevant to this study.

Supplementary Figure S6: The whole blot (uncropped blots) of Figure 5B.

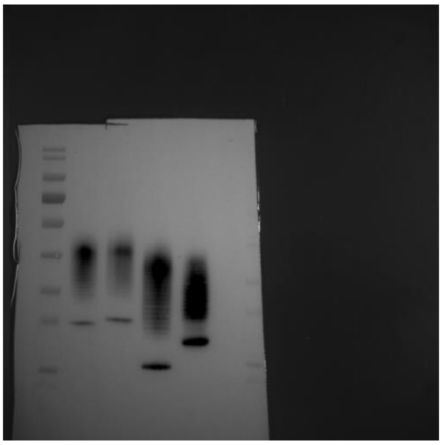

From left to right, lane 1 is protein ladder - from top to bottom - 140, 115, 80, 65, 50, 40, 30, 25, 15, 10 kDa. Lane 3 is nanovaccines NP(LTIB)-OPS_{Kp} and lane 5 is nanovaccines NP(LTIIbB)-OPS_{Kp} pellets were separated by SDS-PAGE and transferred to nitrocellulose membranes for Western Blot analysis using HRP-conjugated anti-6×His tag antibodies. The integrated density of bands was 25603.516 and 77435.014 respectively. Other lanes were not relevant to this study.

Supplementary Figure S7: The whole blot (uncropped blots) of Figure 5B.

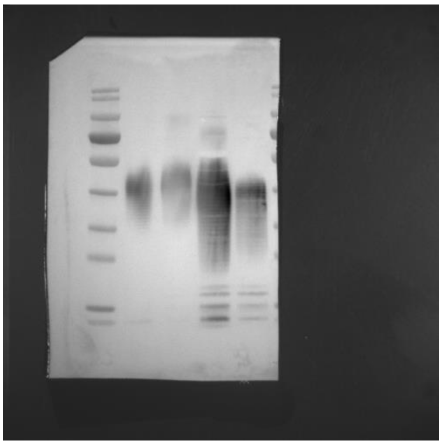

From left to right, lane 1 is protein ladder - from top to bottom - 140, 115, 80, 65, 50, 40, 30, 25, 15, 10 kDa. Lane 3 is nanovaccines NP(LTIB)-OPS_{Kp} and lane 5 is nanovaccines NP(LTIIbB)-OPS_{Kp} pellets were separated by SDS-PAGE and transferred to nitrocellulose membranes for Western Blot analysis using rabbit anti *K. pneumoniae* strain 355 OPS serums. The integrated density of bands was 14625.688 and 26009.597 respectively. Other lanes were not relevant to this study.
